# Psychological mechanisms through which classical tang and song poetry moves contemporary readers: a qualitative study based on the aesthetics of reception

**DOI:** 10.3389/fpsyg.2026.1930677

**Published:** 2026-09-09

**Authors:** Qiqi He, Ruiying Zhang, Zhihao Liang

**Affiliations:** 1 School of Architecture and Urban Planning, Huazhong University of Science and Technology, Wuhan, China; 2 Pan Tianshou College of Architecture and Art Design, Ningbo University, Ningbo, China; 3 School of Architecture and Planning, Hunan University, Hunan, China

**Keywords:** aesthetics of reception, being moved, psychological mechanisms, relational emotions, tang and song poetry

## Abstract

**Background:**

As an important part of classical Chinese literature, Tang poetry and Song ci have long been regarded as having distinctive aesthetic appeal and affect-evoking power. However, existing studies have mostly discussed their artistic value from the perspectives of literary history, textual interpretation, or aesthetic features. Fewer studies have begun from readers' lived experience to examine why contemporary individuals are moved by Tang and Song poetry, or how this experience of being moved is associated with personal experience, relational memory, cultural identity, and life understanding. Therefore, based on the aesthetics of reception and reader-response theory, this study aimed to explore the experiential structure and interpretive psychological processes through which contemporary readers are moved by Tang and Song poetry.

**Methods:**

This descriptive qualitative study conducted semi-structured interviews with 31 adults. The interviews used no standardized list of poems; participants independently recalled and discussed a Tang poem or Song ci that had clearly moved them. Interview data were analyzed at the level of meaning units using inductive content analysis. Categories and subcategories were developed through open coding, cross-case comparison, category aggregation, and category refinement. Independent coding by two researchers, Cohen's kappa, member checking, peer review, negative case analysis, and reflexive memos were used to enhance analytic trustworthiness.

**Results:**

Data analysis identified six categories and 20 subcategories. The categories were sensory aesthetics, individual experience, relational emotions, cultural memory, existential reflection, and barriers and facilitating factors. The findings indicate that textual aesthetic cues provide an initial trigger; personal experience and relational memory establish self-relevance; cultural schemas support understanding; and existential reflection integrates meaning. Facilitating factors and barriers influence these processes through textual accessibility, attentional engagement, and experiential matching.

**Conclusion:**

The power of Tang and Song poetry to move readers is reflected not only in the aesthetic experience evoked by language, imagery, rhythm, and visual scenes, but also in poetry's activation of personal memory, emotional relationships, cultural identity, and life meaning. These findings help explain the contemporary reception of classical Chinese poetry from a psychological perspective. They also suggest that poetry education should prioritize readers' subjective experience by encouraging connections with personal experience, engagement with cultural contexts, and reflection on life.

## 1 Introduction

Research on the psychology of literary reading emphasizes the roles of linguistic form, situational imagination, and self-reference in emotional experience and meaning construction. Existing research shows that artistic and cultural activities have not only aesthetic significance, but may also be associated with emotion regulation, social connectedness, mental health, and subjective wellbeing (Fancourt and Finn, 2019). For example, cultural engagement has been associated with a lower risk of depression (Fancourt and Tymoszuk, 2019), and reading may be related to cognitive maintenance, emotional settling, and a sense of meaning in life (Bavishi et al., 2016). At the same time, literary reading is not merely a process of receiving information. It is also an important way in which readers engage in emotional experience, social imagination, and self-understanding through texts. A recent meta-analysis showed a positive association between fiction reading and social cognition, suggesting that literary texts can activate readers' understanding of others' situations, emotions, and relationships (Dodell-Feder and Tamir, 2018). Therefore, studying why literary works move readers is not only a question for literary studies, but also has clear psychological significance. This study focuses on readers' reported aesthetic, emotional, and meaning-making processes. It does not test clinical outcomes or the effects of health interventions.

In the Chinese cultural context, Tang poetry and Song ci are among the most representative resources of classical literature. They have long been present in school education, family memory, public communication, and everyday expression. They carry aesthetic traditions and also participate in the formation of individual emotional experience and cultural identity. However, existing studies have mostly discussed Tang and Song poetry from the perspectives of literary history, textual interpretation, artistic style, or educational value. Fewer studies have examined the structure and process of this experience of being moved from the subjective perspectives of contemporary readers.

Tang poetry and Song ci are among the most representative and widely circulated poetic texts in classical Chinese literature. Tang poetry organizes language, rhythm, and imagery in highly condensed forms, including five-character verse, seven-character verse, regulated verse, and quatrains, forming an aesthetic tradition characterized by subtlety, leaps in association, blank space, and the fusion of scene and emotion (Cai and Wu, 2019; Fuller, 2018). Song ci developed from musical tune structures and, through tune patterns, lines of varying length, tonal prosody, and rhythmic change, became especially suited to expressing delicate emotions, private experience, and complex relational feelings (Fuller, 2013; Zhu, 2025). From the perspective of literary history, Tang poetry and Song ci not only represent aesthetic peaks of different dynasties, but also reflect the continuous expansion of Chinese poetry from literati self-expression, landscape inspiration, frontier separation, and parting to urban experience, individual emotion, and reflection on life (Owen, 2016; Egan, 2006).

The long-standing presence of Tang and Song poetry in Chinese education, everyday expression, and cultural memory is also related to their highly condensed emotional structure. Scholars have noted that friendship, farewell, political frustration, and landscape experience in Tang poetry are often closely connected with literati identity and social relationships (Shields, 2015), whereas Song ci further intensified delicate and subjectivized expressions of emotion in themes such as love, longing, mourning, travel, and concern for family and country (Egan, 2013; Ridgway, 2012). In recent years, digital humanities research has also shown, from the perspectives of vocabulary, color, imagery, and semantic patterns, that Tang and Song poetry display identifiable historical differences and aesthetic features in color terms, natural imagery, and emotional expression (Liu, 2016; Liu and Luo, 2016; Liu, 2019; Gong and Zhou, 2025). This suggests that Tang and Song poetry is not merely an ancient literary heritage, but a body of classical texts that combines formal beauty, emotional density, cultural memory, and psychological evocation. Thus, taking Tang and Song poetry as the object of study helps examine how literary reading activates aesthetic perception, personal experience, relational emotions, and cultural identity through indigenous Chinese cultural materials.

From the perspective of aesthetic psychology, literary reception is not a passive reception of textual meaning, but a psychological process in which aesthetic perception, emotional arousal, cognitive appraisal, self-related experience, and cultural identity jointly participate. Early experimental aesthetics understood aesthetic experience as a subject's perception and evaluation of an object's formal features (Fechner, 1876; Berlyne, 1971). Later models of aesthetic processing further suggested that aesthetic judgment usually involves stages such as perceptual analysis, memory integration, meaning interpretation, emotional evaluation, and aesthetic judgment (Leder et al., 2004; Leder and Nadal, 2014). Within this framework, the linguistic rhythm, imagery structure, sound patterns, and visual quality of Tang and Song poetry can be understood as textual cues that trigger aesthetic perception and emotional response. Recent research in neuroaesthetics and empirical aesthetics has further emphasized that aesthetic experience involves not only sensory pleasure, but also emotional involvement, meaning generation, and self-referential processing (Chatterjee and Vartanian, 2014; Vessel et al., 2012; Belfi et al., 2018). Literary texts are especially different from visual art in that they evoke imagination, memory, and situational simulation through linguistic signs. Foregrounding theory and neurocognitive poetics argue that defamiliarization, rhythm, metaphor, and structural deviation in literary language can slow automatic understanding and prompt deeper attentional, affective, and meaning processing (van Peer, 1986; Miall and Kuiken, 1994; Jacobs, 2015). This view resonates with the aesthetics of reception, which emphasizes readers‘ participation in meaning generation. Meanwhile, research on aesthetic emotions shows that being moved, awe, nostalgia, sadness, tranquility, and complex mixed emotions in art reception often constitute deeper experiences than simple pleasure (Silvia, 2005, 2009; Schindler et al., 2017). In the case of Tang and Song poetry, readers are moved not only by linguistic beauty, but also by connections between the text and personal memory, social relationships, and cultural identity. Cultural psychology has shown that individuals' self-understanding and emotional expression are deeply shaped by cultural models (Vignoles et al., 2016). Therefore, studying the reception of Tang and Song poetry from the perspective of aesthetic psychology can help explain how classical poetry activates aesthetic perception, emotional experience, cultural identity, and life meaning among contemporary readers.

Existing research has revealed the artistic value of Tang and Song poetry from the perspectives of literary history, textual interpretation, poetry education, and aesthetic theory, and has also shown from aesthetic psychology that literary reception involves multiple psychological processes, including perception, emotion, memory, selfhood, and culture. However, current research on why Tang and Song poetry moves people remains insufficient. On the one hand, relevant discussions often remain at the level of textual aesthetics or literary education, and seldom systematically examine the psychological process of being moved from the subjective experience of contemporary readers. On the other hand, psychological research has focused mainly on general literary reading, artistic aesthetics, or fictional narrative, with insufficient attention to classical Chinese poetry as a highly condensed, image-rich, and culturally loaded text type. It is therefore necessary to take contemporary readers as the research participants and further explore how Tang and Song poetry evokes aesthetic experience, emotional resonance, and meaning construction in actual reception.

Drawing on the aesthetics of reception, reader-response theory, and aesthetic psychology, this study adopts the following analytical framework. The power of Tang and Song poetry to move contemporary readers does not derive from a single textual factor. Instead, it emerges through several interrelated experiential domains, including sensory aesthetics, individual experience, relational emotions, cultural memory, and existential reflection.

Facilitating factors and barriers influence this process by altering textual accessibility, attentional engagement, and experiential matching. Iser's (1978) concepts of the “structure of appeal” and “gaps” explain how textual cues invite readers to complete meaning. Jauss (1982) 'horizon of expectations' explains how education and cultural knowledge shape reading expectations. Rosenblatt (1978) transactional view of reading explains how text and reader experience jointly generate meaning in a specific reading event. These perspectives are complementary rather than merely juxtaposed: Iser focuses on textual openness, Jauss on readers' prior cultural expectations, and Rosenblatt on text-experience transactions within the reading event. Aesthetic psychology connects perception, emotion, memory, and meaning construction. These theories served as sensitizing frameworks for interview design and interpretation, not as a predefined coding template. Given the qualitative design, we describe these relations as an 'interpretive psychological process.' This term refers to subjective experiences and conditional relations inferred from participant narratives and interpreted theoretically, rather than to a causal mechanism validated through experimental manipulation.

Accordingly, the central psychological research question of this study is: through which cognitive and affective pathways does Tang and Song poetry evoke aesthetic experience among contemporary readers? To answer this question, this study used semi-structured interviews and content analysis to examine contemporary readers' aesthetic feelings, emotional responses, associations with personal experience, relational emotions, cultural memory, and life understanding when reading Tang and Song poetry, and further identify the experiential structure and interpretive psychological processes through which Tang and Song poetry moves readers.

## 2 Methods

### 2.1 Study design

This study was reviewed by the relevant ethics review body of the School of Architecture and Planning, Hunan University, which provided written confirmation. The study involved low-risk, non-interventional, anonymous interviews and collected no clinical intervention data or sensitive physiological data. Audio recordings and narrative materials were de-identified during transcription and managed in accordance with informed-consent and confidentiality requirements. All participants provided written informed consent before the interviews, and the research followed the relevant principles of the Declaration of Helsinki.

This study used semi-structured, one-to-one, in-depth interviews to conduct a descriptive qualitative study in Central, Eastern, and Southern China from March to May 2026, and extracted categories and subcategories through content analysis. Purposeful sampling and maximum variation sampling were used to select participants so as to cover, as far as possible, contemporary readers with different ages, genders, educational backgrounds, occupational experiences, degrees of familiarity with poetry, and reading experiences. The researchers released recruitment information through universities, community cultural activity venues, online social platforms, and acquaintance referrals, and explained the research purpose, interview content, data use, and confidentiality principles to potential participants. Before each formal interview, the researchers again explained that the study mainly concerned participants' subjective feelings, emotional experiences, and personal understanding when reading Tang and Song poetry, confirmed voluntary participation, and obtained informed consent. Both Tang poetry and Song ci were included because the study sought to identify shared processes in contemporary reception of classical Chinese poetry, rather than compare genre effects. The analysis nevertheless recorded and retained the genre and specific context of each work discussed by participants. Here, 'shared process' denotes recurring experiential structures across narratives; it does not assume that Tang poetry and Song ci are unaffected by genre differences.

Participants were included if they met the following criteria: (i) aged 18 years or older; (ii) able to express themselves and communicate clearly in Mandarin Chinese; (iii) had received basic education in Tang poetry and Song ci, or had experience reading, reciting, studying, or encountering poetry in everyday life; (iv) were willing to participate in an in-depth interview about reading experience, personal experience, relational emotions, cultural memory, and life feelings related to Tang and Song poetry; and (v) voluntarily participated in the study and signed an informed consent form. Participants were excluded if they met any of the following criteria: (i) had cognitive, hearing, or communication impairments that clearly affected verbal expression or interview communication; (ii) were unable to complete the interview for personal reasons; (iii) refused audio recording or refused the use of their interview data for research analysis; or (iv) their interview content clearly deviated from the research topic and could not provide valid information related to the reception experience of Tang and Song poetry.

### 2.2 Data collection

After a comprehensive literature review and in-depth team discussion, the interview guide was finalized (Table 1). Before each interview, the researchers fully explained the study details and obtained informed consent after confirming participants' understanding. All participants were informed that they could withdraw at any time without providing a reason. Face-to-face interviews were conducted in private spaces at community service centers or participants' homes. Online video or voice interviews and telephone interviews were conducted remotely using the corresponding modes. A trained doctoral student with substantial experience in qualitative research conducted all interviews. With participants' consent, all interviews were audio-recorded. The researcher documented observable facial expressions and nonverbal behavior during face-to-face and video interviews; unobservable visual information was not recorded during voice or telephone interviews. Each participant was anonymized and assigned a code to protect confidentiality. The research team assessed saturation as interviewing and preliminary coding proceeded concurrently. Categorical saturation was reached when consecutive interviews yielded no new subcategories and did not alter existing category definitions (Hennink and Kaiser, 2022). This criterion was met after the 24th interview, and seven additional participants were interviewed for confirmation. Data from 31 participants were included in the final analysis.

**Table 1:** Semi-structured interview guide.

Interview questions	Interview focus (for open-ended probing only; not a predefined coding category)
Have you ever been moved by a Tang poem or Song ci? Please specify which poem or line it was and describe how you felt at the time.	Emotional arousal; subjective aesthetic experience
Why do you think it moved you? Was it because of language, rhythm, visual scene, imagery, emotion, or other reasons?	Aesthetic perception; cognitive appraisal
Did this poem or lyric remind you of your own experiences, circumstances, or feelings about a particular stage of life? Please describe this in detail.	Autobiographical memory; self-referential processing
Did this poem or lyric remind you of a person, a relationship, or a particular interpersonal emotion? Why?	Social connectedness; relational memory
Do you think your educational experience, cultural memory, or familiarity with traditional culture influences whether you are moved by this poem or lyric?	Cultural schemas; collective memory; cultural identity
Did this poem or lyric give you any understanding or comfort concerning life, time, gain and loss, parting, loneliness, or fate?	Meaning construction; existential reflection
What factors do you think strengthen or weaken the power of Tang poetry or Song ci to move you?	Reception conditions; cognitive accessibility; emotional involvement

The interviews used no standardized list of poems or common stimulus. Participants independently selected a Tang poem or Song ci that had clearly moved them and, where possible, identified the title, key lines, feelings at the time, and relevant context. Works were not screened by prominence, topic, or author. Because the unit of analysis was the meaning unit across narratives about different works, rather than response differences to a common poem, participants did not need to discuss the same poem.

The interview guide followed a theory-sensitized, open-probing approach. Questions about language, action, imagery, and gaps addressed the textual structure of appeal. Questions about personal experience and relational memory addressed transactional reading. Questions about education, traditional imagery, and historical knowledge addressed the horizon of expectations. Aesthetic psychology provided process-oriented reference points for emotional arousal, understanding of life, and facilitating or impeding conditions. These correspondences ensured adequate coverage in open-ended probing; they were not used as predefined coding categories.

### 2.3 Data analysis

This study used content analysis to organize and analyze the interview data. After each interview, the researchers promptly transcribed the audio recordings verbatim and checked the transcripts against the interview records to ensure data completeness and accuracy. All personally identifiable information was anonymized during transcription and data organization.

The specific analytical process was as follows. First, the researchers repeatedly read the interview transcripts to obtain an overall understanding of participants‘ narratives about being moved by Tang and Song poetry, and marked meaning units related to the research question. Second, open coding was conducted on the meaning units to extract initial codes that reflected readers' aesthetic feelings, personal experiences, relational associations, cultural memories, and life understanding. The researchers then compared similarities and differences among codes, aggregated codes with similar meanings into subcategories, and further grouped these into higher-level categories. Finally, six categories and 20 subcategories were formed, including sensory aesthetics, individual experience, relational emotions, cultural memory, existential reflection, and barriers and facilitating factors. The six categories were inductively derived from the interview data. The theories described above informed the interview guide and the interpretation of categories after coding, but were not used as an initial code list or to force data into predetermined categories.

All interview recordings were transcribed verbatim and checked against the audio recordings and interview notes. Data analysis used the meaning unit as the basic unit of coding, defined as a sentence, short paragraph, or narrative fragment that independently expressed an aesthetic feeling, emotional response, personal experience, relational association, cultural memory, or life understanding. The analysis combined manual coding with tabular data management. Before any consensus discussion, two coders independently classified the same set of meaning units using the same codebook. Cohen's kappa was calculated separately for category and subcategory assignment: kappa = 0.89 at the category level and kappa = 0.86 at the subcategory level, indicating high inter-coder agreement for the established codebook. For disagreements, the research team returned to the original context, discussed the cases, and refined the code definitions until consensus was reached. Kappa assessed whether the two coders assigned the same meaning units to the same categories and subcategories; it did not quantify participants or their experiences. It was used only to assess code boundaries and coding stability, not for hypothesis testing, effect estimation, or statistical inference. Its use therefore does not make the study a mixed-methods design.

To enhance the trustworthiness of the study, member checking, peer review, and negative case analysis were used. Some interview summaries and preliminary interpretations were returned to participants to confirm whether the researchers‘ understanding was consistent with participants' intended meaning. Researchers with experience in qualitative research and literary studies were also invited to review the coding framework and category labels. The researchers paid particular attention to negative cases, such as materials in which participants were not moved by poetry, experienced aesthetic dulling because of excessive familiarity, or found allusions difficult to understand because of cultural distance, and incorporated these materials into the analysis. Because the researchers themselves had experience reading classical literature, which might influence data interpretation, the research team used analytic memos to record the interpretive process and reflect on its own presuppositions, thereby enhancing transparency and traceability.

## 3 Results

### 3.1 Sociodemographic characteristics

A total of 31 participants were included in the formal analysis, including 24 face-to-face interviews, five online video or voice interviews, and two telephone interviews. The mean interview duration was 46.3 + /− 7.8 min, with a range of 32.00 to 60.00 min. Participants' demographic characteristics are shown in Table 2.

**Table 2:** Demographic characteristics.

Characteristics		*n*	%
Age (Mean ± SD, min to max, years)		32.5 ± 8.8 (20.00 to 55.00)	
Gender	Female	16	51.61%
	Male	15	48.39%
Participant identity/occupation	Cultural/media/writing	6	19.35%
	Student	6	19.35%
	Healthcare/psychological service	4	12.90%
	Teacher/education worker	3	9.68%
	Business/management	2	6.45%
	Public service	2	6.45%
	Technology/engineering	2	6.45%
	Administration/service	2	6.45%
	Legal profession	1	3.23%
	Sales/service	1	3.23%
	Self-employment/freelance	1	3.23%
Primary text type discussed	Tang poetry	16	51.61%
	Song poetry	15	48.38%
Interview style	Face-to-face	24	77.42%
	Online video/voice interview	5	16.13%
	Telephone interview	2	6.45%
Interview time (Mean ± SD, min to max, minutes)		46.3 ± 7.8 (32.00 to 60.00)	
Data saturation	Reached after the 24th interview		

**Figure 1:**
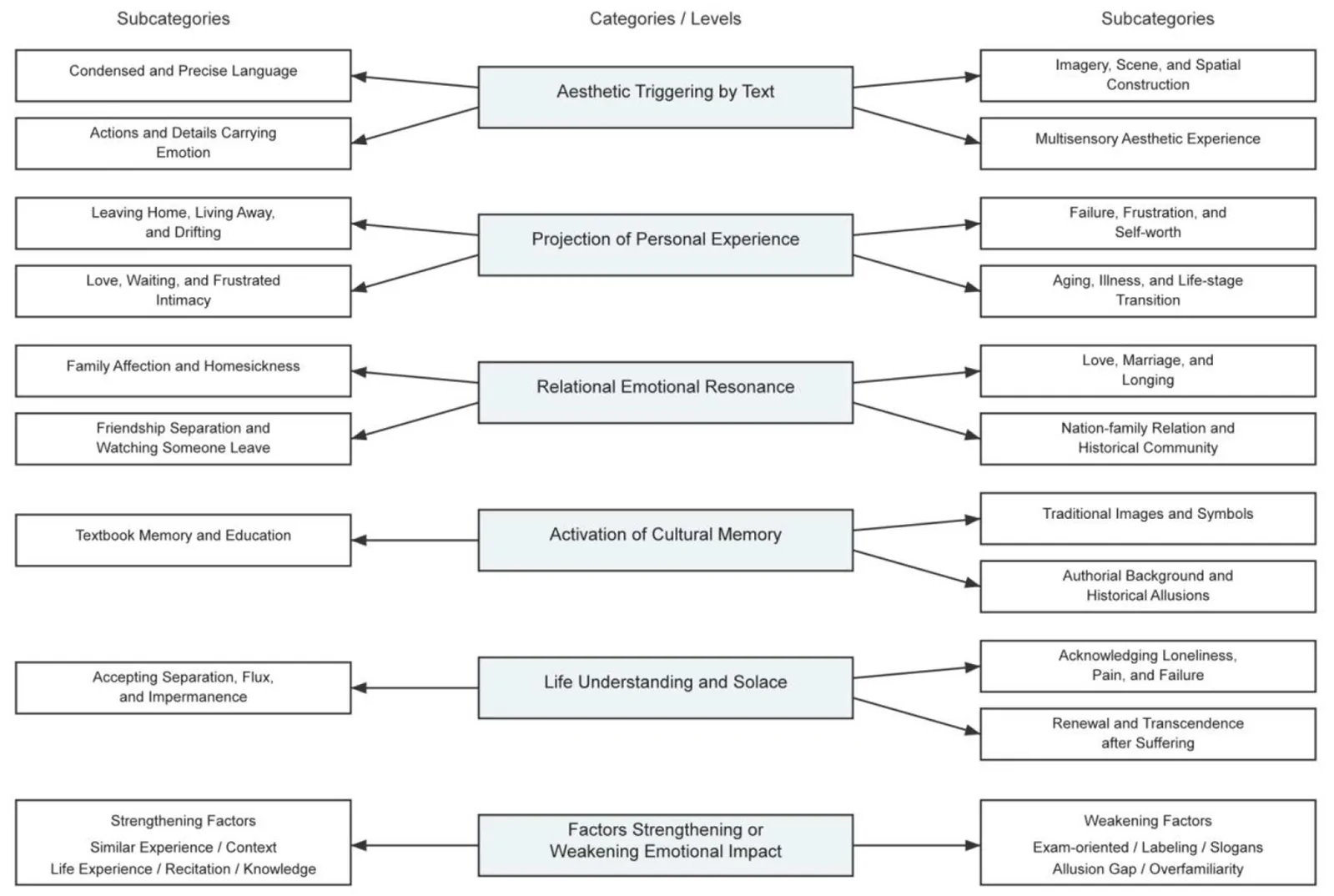
Six experiential categories and 20 subcategories through which classical Tang and Song poetry moves contemporary readers.

Six categories were extracted from the interview data: (i) textual aesthetic triggers, including condensed and precise language, actions and details carrying emotion, imagery, visual scenes and spatial construction, and multisensory aesthetic experience; (ii) projection of individual experience, including leaving home, living elsewhere and drifting, love, waiting and frustration in intimate relationships, failure, frustration and impaired self-worth, and aging, illness and life-stage transitions; (iii) relational emotional resonance, including family affection and attachment to hometown, the dispersal of friendship and relationships of watching someone depart, love, marriage and longing, and relations between family and country and a historical community; (iv) activation of cultural memory, including textbook memory and educational activation, traditional imagery and cultural symbols, and authorial background and historical allusions; (v) life understanding and spiritual settling, including accepting parting, flow and impermanence, acknowledging loneliness, suffering and failure, and renewal after hardship and open-minded transcendence; and (vi) barriers and facilitating factors (conditions of reception), including facilitating and weakening factors, as shown in Figure 1. The six categories are data-derived experiential categories, and Figure 1 depicts their category-subcategory structure. In the Discussion, they are integrated into an interpretive process comprising aesthetic triggering, experiential connection, relational and cultural mediation, meaning construction, and conditional modulation. Each major category was supported by multiple participants and materials from multiple subcategories. Given space constraints, the quotations illustrate category content rather than serve as statistical proof. Categories were established through recurrence across all relevant meaning units, cross-case comparison, and negative case analysis.

### 3.2 Category 1: Sensory aesthetics

The interviews showed that many participants did not initially understand poetry at the level of life philosophy or cultural meaning. Instead, they were first touched by a particular word, action, visual scene, sound, smell, or sense of space. In other words, the moving power of Tang and Song poetry first appeared as an aesthetic entry point: readers could see, hear, and feel the scenes in the poems, and thereby developed an initial emotional response.

#### 3.2.1 Subcategory 1: Condensed and precise language

Condensed and precise language was a reason participants mentioned for being moved. Participants believed that Tang and Song poetry is moving not because its wording is complex, but because it can accurately express, in very few words, emotions that are difficult to articulate. For example, when discussing Quiet Night Thoughts by Li Bai, P04 stated:

“It captures a universal action: being alone at night, seeing the moon, and then missing home. ‘Raising the head' and ‘lowering the head' are especially good, one looks outward, and the other returns inward.”

This shows that the participant did not simply understand Quiet Night Thoughts as a poem of homesickness, but noticed the psychological shift behind the two actions of raising and lowering the head. The simplicity of the language instead strengthens the universality of the emotion, allowing readers to quickly enter the poem's situation.

Similarly, when discussing Climbing the Heights by Du Fu, P11 noted:

“It presses many kinds of suffering together: drifting, autumn, illness, aging, and loneliness. The language is dense, as if half a lifetime were packed into a single line.”

This indicates that condensed language can carry complex life experiences. The short lines in poetry are not semantically poor, but highly compress multiple emotions and life situations.

#### 3.2.2 Subcategory 2: Actions and details carrying emotion

In addition to language itself, concrete actions and details were also important factors that elicited being moved. Participants generally believed that some seemingly simple actions in poetry, such as urging someone to drink, watching someone leave, holding hands, and lowering the head, can carry rich and implicit emotions.

When discussing Wang Wei's Sending Yuan Er on a Mission to Anxi, P01 said:

“It does not cry or shout. It only writes about urging someone to drink, but that action is weighty. Sometimes Chinese people do not say, “I cannot bear to part with you,” but say, “Eat a little more” or “Take care on the road.””

Here, the line “I urge you to finish one more cup of wine” is moving because it transforms emotions that are difficult to state directly at parting into a concrete action. The participant connected the ancient farewell act of urging someone to drink with modern experiences of seeing someone off at a station and giving parting reminders, suggesting that action details can cross historical time and activate readers' own life experiences.

P03 also mentioned Liu Yong's Yulin Ling:

“When people are extremely sad, language really disappears. Holding hands, looking at each other, tearful eyes, no words, choking with sobs, every action is concrete.”

This suggests that poetry moves readers not only through abstract lyricism, but through embodied emotional details that readers experience as authentic and credible. Being “wordless and choking with sobs” is not a conceptualized sadness, but a bodily reaction that may truly occur at the scene of parting, and is therefore more likely to evoke resonance.

#### 3.2.3 Subcategory 3: Imagery, visual scenes, and spatial construction

The imagery, visual scenes, and spatial structures in Tang and Song poetry had marked appeal for readers. When many participants described why they were moved, they first described what they “saw” in the poem.

When discussing Li Bai's Seeing Meng Haoran off to Guangling at Yellow Crane Tower, P02 stated:

“Yellow Crane Tower, the Yangtze River, the blue sky, and a lone sail. The space is vast, but the human feeling is delicate. It does not directly write that one cannot bear to part, but writes the boat moving farther and farther away.”

This indicates that space in poetry is not merely background, but an amplifier of emotion. The vast river and sky, together with the lone sail gradually disappearing, express the emotion of parting through visual distance. Participants were moved not by a single image, but by a spatial experience jointly constituted by multiple images.

P10 also noted Chen Zi'ang's Song on Climbing Youzhou Terrace:

“The larger heaven and earth become, the lonelier the person feels. ‘Before me I see no ancients; behind me I see no successors' feels as if a person's value is held by neither the past nor the future.”

Here, being moved arose from a vast sense of time and space. The poem's “before,” “behind,” and “heaven and earth” create temporal and spatial depth. Placed within this depth, the individual experiences loneliness in a strongly magnified form. Thus, Tang and Song poetry often gives abstract emotion a perceptible form through visual scenes and spatial structures.

#### 3.2.4 Subcategory 4: Multisensory aesthetic experience

Participants also mentioned multisensory experiences, including sound, smell, touch, and overall atmosphere. Such feelings often appeared in landscape and pastoral poetry, giving readers a quiet, soothing, or healing aesthetic experience.

When discussing Wang Wei's Autumn Evening in the Mountains, P13 said:

“The picture and the sound are both clear. Bright moon, pine forest, clear spring, stones, these images have no impurities. For me, it is like a kind of mental noise reduction.”

This indicates that natural images in poetry not only form visual scenes, but also bring a sense of auditory and psychological quietness. Images such as the bright moon, pines, clear spring, and stones create a lucid and tranquil aesthetic space, allowing readers to find a brief settling amid the pressures of modern life.

When discussing Xin Qiji's Xijiang Yue: Walking at Night on the Yellow Sand Road, P15 stated:

“The fragrance of rice flowers and the sound of frogs are not abstract beauty. They can be smelled and heard.”

This statement shows that the moving power of Tang and Song poetry comes not only from semantic understanding, but also from the awakening of sensory memory. The smell of rice flowers and the auditory experience of frogs connect the text with readers' childhood memories, rural experience, and bodily feelings. Multisensory aesthetics means that poetry is no longer only an object to be understood, but becomes a life scene that can be re-experienced.

### 3.3 Category 2: Individual experience

The interviews showed that many participants were not moved when they first learned a poem, but only after they had experienced leaving home, drifting, romantic frustration, failure, illness, or aging did they understand the emotions in the poem anew. In other words, the moving power of poetry comes not only from the text itself, but also from the participation of readers' life experiences. Readers often transform originally familiar lines into part of their personal experience through a process of “understanding only later.”

#### 3.3.1 Subcategory 1: Leaving home, living elsewhere, and drifting

Experiences of leaving home, living elsewhere, and drifting were among the individual experiences most frequently mentioned by participants. Many participants stated that when they recited homesickness poems in childhood they felt little depth, but only after they truly left their hometown and lived alone elsewhere did they understand the feelings of being away from home and searching for belonging in the poems.

When discussing Fan Zhongyan's Su Muzhe: Nostalgia, P06 mentioned:

“After middle age, drifting is not romantic. It is exhausting.”

The sense of drifting is not simply homesickness. It also includes long-term mobility, life pressure, and middle-aged fatigue. For such participants, travel sorrow in poetry is not abstract lyricism, but overlaps with real experiences such as business trips, unfamiliar cities, and being alone at night.

#### 3.3.2 Subcategory 2: Love, waiting, and frustration in intimate relationships

Loss in love, waiting, and intimate relationships was also an important experiential source of being moved by poetry. The interviews showed that readers often transformed boudoir resentment, parting, and longing in classical poetry into modern romantic experience, long-distance relationships, or the psychological state of waiting for a message.

When discussing Wen Tingyun's Wang Jiangnan: After Dressing, P08 said:

“Modern people may not watch boats from upstairs, but they keep looking at their phones. Even though they know there may be no result, they cannot help refreshing.”

This indicates that the experience of waiting in classical poetry can be reinterpreted by modern readers. “After a thousand sails have passed, none is the awaited one” is no longer only a scene of an ancient woman leaning from a tower and looking into the distance. It can also correspond to the psychology of modern people repeatedly checking their phones and waiting for a reply. The moving power of poetry is activated precisely in this transformation between ancient and modern experience.

#### 3.3.3 Subcategory 3: Failure, frustration, and impaired self-worth

Some participants were easily moved, during career lows, academic failure, or life setbacks, by poems that express unrecognized talent, thwarted ambition, and life oppression. This kind of being moved did not arise entirely from sadness, but from the poem's precise expression of “not having one's value seen.”

When discussing Xin Qiji's Broken Array: A Heroic Lyric for Chen Tongfu, P18 mentioned:

“The hardest thing is not losing money, but realizing that what you wanted to do has not been achieved, and that much passion has eventually turned into fatigue.”

This suggests that the poetic theme of “thwarted ambition” can connect with modern experiences of entrepreneurial failure and frustrated ideals. The key to being moved was not only the heroic spirit in the lyric, but the sudden fall from grandeur into the powerlessness of reality. Poetry here provides an expressive form for experiences of failure, allowing individual frustration to become not only a private predicament, but an intelligible emotional structure.

#### 3.3.4 Subcategory 4: Aging, illness, and life-stage transitions

Aging, illness, and life-stage transitions were also important factors that activated the moving power of poetry. Some participants stated that when they were young, they found it difficult to understand certain somber, sorrowful, or open-minded poems. Only after experiencing bodily change, the aging of relatives, or life-stage transitions did they resonate deeply with them.

When discussing Liu Yuxi's Replying to Lotte at the First Meeting in Yangzhou, P22 stated:

“When I was ill, I felt I had fallen behind while everyone else was moving forward. Only after recovering did I realize that life is not defined by that period of stagnation.”

This quotation shows that the experience of illness not only made readers feel vulnerable, but also enabled them to better understand the poem's sense of renewal. The line “before the diseased tree, ten thousand trees are in spring” moves people because it acknowledges the existence of damage while also offering the possibility of beginning again. For those who have experienced illness and recovery, poetry provides not only an aesthetic experience, but also a form of comfort about life-stage transition.

### 3.4 Category 3: Relational emotions

An important reason Tang and Song poetry moves readers is not simply that it expresses isolated personal emotion, but that it places the individual within relationships of family affection, friendship, love, marriage, and family-country belonging. Through poetry, readers often re-understand their own attachment, separation, longing, and responsibility in relation to others. Therefore, relational emotions constitute an important mediator of the moving power of Tang and Song poetry.

#### 3.4.1 Subcategory 1: Family affection and attachment to hometown

Family affection and attachment to hometown were among the relational emotions with which participants most easily resonated. The interviews showed that when many participants discussed homesickness poems, they did not think only of hometown in a geographical sense, but also of parents, family members, and family life.

P26 said:

“Missing one's hometown is actually missing one's parents. No matter how far a person goes, there is still a place in the heart that holds them.”

This shows that family attachment gives homesickness a durable quality. Even if participants had left their hometown for a long time, poetry could still evoke, at particular moments, their attachment to parents and family. Thus, homesickness in Tang and Song poetry can cross historical time because it transforms spatial distance into relational attachment.

#### 3.4.2 Subcategory 2: The dispersal of friendship and relationships of watching someone depart

The dispersal of friendship was also an important relational experience through which Tang and Song poetry moved participants. Unlike the sense of belonging in family affection, being moved in friendship often appeared as the feeling of watching someone depart during graduation, long journeys, parting, and the gradual distancing of relationships.

When discussing Li Bai's Seeing Meng Haoran off to Guangling at Yellow Crane Tower, P02 said:

“True friendship can also mean watching someone go. It does not necessarily mean holding them back.”

This quotation shows that the participant did not understand farewell as mere sadness, but as a relational way of respecting the other's journey. The poem's “distant shadow of the lone sail” expresses not only the friend's departure, but also the emotional posture of the one who remains in place and watches the friend enter the distance.

P25 also mentioned graduation parting:

“Graduation parting is still lively at the moment. The real sadness comes when you return to the empty dormitory and find that someone's bed is empty.”

This suggests that the pain of friendship dispersal does not always appear immediately at the scene of parting, but often emerges later in the absence left in everyday life. Farewell imagery in Tang and Song poetry enabled participants to re-understand the relational experience of “gradually moving farther apart” in modern friendship.

#### 3.4.3 Subcategory 3: Love, marriage, and longing

Love, marriage, and longing were important entry points for participants' understanding of Song ci and some Tang poems. The interviews showed that participants often connected longing, waiting, and parting in classical poetry with modern romantic relationships, long-distance relationships, marital separation, and everyday attachment.

When discussing Li Qingzhao's A Spray of Plum Blossoms, P09 said:

“Longing is not one person's unilateral sadness, but something two people bear together in different places.”

This statement shows that the participant understood longing as a bidirectional relationship rather than one-sided emotional depletion. The line “one kind of longing, two places of idle sorrow” is moving precisely because it allows two separated people to remain emotionally connected.

P27 also commented on longing in marriage:

“Longing after marriage is not necessarily romantic. It is often mixed into housework, children, and waiting for a phone call.”

This quotation suggests that love in classical poetry can be reinterpreted through modern married life. Poetry presents these small and often inexpressible attachments, making readers feel that their own experience has been seen.

#### 3.4.4 Subcategory 4: Family-country relations and historical community

Beyond private relationships, some participants also felt an emotional connection between the individual and the country, and between the family and history, in poems of family-country concern. The interviews showed that participants were more likely to be moved by concrete family-country emotion than by abstract slogan-like expression.

When discussing Du Fu's Spring View, P16 said:

“Family and country are not abstract. Turmoil ultimately falls on every family.”

This statement indicates that when participants understood concern for family and country, they did not remain at the level of grand narrative, but connected national upheaval with concrete experiences such as family separation and loss of contact with relatives. The line “a letter from home is worth ten thousand pieces of gold” is moving precisely because it transforms national crisis into the immediate pain of ordinary families.

P31 also noted:

“What truly moves people in family-country feeling is not shouting slogans, but a person's cherishing of loved ones, home, and order.”

This shows that the moving power of family-country relations comes from their connection with everyday life and intimate relationships. Family-country concern in Tang and Song poetry is not detached from personal emotion, but is expressed through concrete objects such as loved ones, home, order, and a sense of safety. Thus, what readers feel in poetry is not only a historical event, but a transhistorical emotion of community.

### 3.5 Category 4: Cultural memory

The moving power of Tang and Song poetry for contemporary readers does not come entirely from immediate reading experience. It is also related to readers' long-accumulated cultural experience. Many participants mentioned that some poems had entered their memory as early as primary or secondary school, but at that time existed only as material for recitation or examination. Only later, when they encountered similar life situations, were the familiar lines reactivated. Thus, cultural memory can both enhance the moving power of poetry and obscure its genuine emotion through exam-oriented and label-based modes of reception.

#### 3.5.1 Subcategory 1: Textbook memory and educational activation

Textbook memory was an important precondition for participants' understanding of Tang and Song poetry. Many participants stated that their first contact with poetry mainly came from school education. Although they were not necessarily moved at that time, these poems were stored for a long time as cultural memory and were reactivated by later life experiences.

When discussing Sending Yuan Er on a Mission to Anxi, P01 said:

“What I memorized as a child was the standard answer. Later, when I encountered a similar scene, the poem would surface by itself.”

This statement indicates that school education allows poetry to enter readers' memory in advance, but genuine being moved often occurs in later life scenes. When participants experienced leaving home or parting, lines that had originally existed as knowledge points were transformed into tools for expressing personal experience.

P04 also mentioned:

“Education stores it first, and life experience activates it later.”

This suggests that educational experience does not simply weaken readers' feelings for poetry. The key is whether it becomes connected with later life experience. Textbook memory provides readers with a storehouse of poetry that can be awakened, while life experience gives these lines renewed emotional warmth.

#### 3.5.2 Subcategory 2: Traditional imagery and cultural symbols

Traditional imagery and cultural symbols were important media through which Tang and Song poetry moved readers. The interviews showed that readers' familiarity with images such as the bright moon, willows, the waning moon, autumn scenes, peach blossoms, and swallows affected their understanding of poetic emotion. Such images not only form visual scenes, but also carry long-accumulated cultural meanings.

When discussing Liu Yong's Yulin Ling, P03 said:

“Knowing some traditional imagery deepens the feeling. Willows and the waning moon are not decorations, but vessels of emotion.”

This statement shows that traditional imagery helps readers enter the poetic situation. The cultural associations among willows and parting, the waning moon and coldness, and the morning wind and post-parting loneliness mean that readers do not merely see scenery, but feel the emotions sedimented within it.

P19 also stated, when discussing Cui Hu's Written at South Village, the Capital City:

“The world remains beautiful as usual, but the person you wanted to see is gone. The word ‘as before' is cruel.”

Here, peach blossoms are not merely spring scenery, but a cultural symbol of things remaining while people have changed. The continued blooming of peach blossoms highlights the person's absence and the irreversibility of time. Thus, the moving power of traditional imagery comes from its multiple meanings: it is both a concrete and perceptible scene and a cultural symbol carrying time, relationships, and loss.

#### 3.5.3 Subcategory 3: Authorial background and historical allusions

In addition to textbook memory and traditional imagery, knowledge of the poet's life, historical background, and allusions also affected readers' experience of being moved. Some participants stated that when they understood the real predicament in which a poet lived, the emotion in the poem became more credible and weighty.

When discussing Su Shi's Busuanzi: Written at Dinghui Temple in Huangzhou, P12 said:

“Knowing the background of Su Shi's banishment to Huangzhou is important. Otherwise, one might only feel that this is a cold and quiet picture.”

This quotation shows that background knowledge can change the level of readers' understanding. Without knowledge of Su Shi's banishment, the lone wild goose, cold branches, and sandbank might be only cold and lonely images. With that background, these images become symbols of personal circumstance and spiritual self-possession.

P29 also mentioned Liu Yuxi's Wuyi Lane:

“Without the allusion to Wang and Xie, half of the moving power would be lost. Cultural knowledge here is not a burden, but the key that opens the poem.”

This indicates that the moving power of some poems depends strongly on historical allusion. Only when readers understand the family status, prosperity, and historical rise and fall represented by “Wang and Xie” can they truly read the sense of time behind “flying into the homes of ordinary people.” Cultural knowledge therefore does not necessarily create a barrier to reading. On the contrary, when combined with readers' experience, it can expand the meaning space of poetry and deepen emotional understanding.

### 3.6 Category 5: Existential reflection

The interviews showed that some poems continue to move readers because they can transform life experiences that individuals find difficult to express into forms that can be understood, acknowledged, and settled. In poetry, readers not only feel moved, but also feel that their situation has been explained, confirmed, or even comforted.

#### 3.6.1 Subcategory 1: Accepting parting, flow, and impermanence

Parting, relational flow, and the impermanence of time were life themes repeatedly felt by participants in poetry. Unlike simple sadness, some participants stated that poetry helped them understand the distancing of people from one another, changes in relationships, and the fact that time is irreversible.

When discussing Written at South Village, the Capital City, P19 stated:

“The world goes on as usual, and that is what makes it saddest.”

This quotation reveals the deep moving power of temporal impermanence. Things remaining while people have changed is shocking not only because the person has left, but because the world does not stop for an individual's loss. The peach blossoms continue to bloom and the spring wind continues to blow. It is precisely this “as usual” that highlights the irreversibility of time and human powerlessness. Poetry here helps readers understand that impermanence does not always appear in dramatic form. It is often hidden in the seemingly calm continuation of everyday life.

#### 3.6.2 Subcategory 2: Acknowledging loneliness, suffering, and failure

The interviews showed that Tang and Song poetry can also help readers acknowledge loneliness, suffering, and failure. Some participants did not expect poetry to provide direct solutions. Instead, they believed that poetry's important value lies in accurately speaking pain, making individuals feel that their situation is understood.

When discussing Song on Climbing Youzhou Terrace, P10 said:

“Loneliness sometimes comes from questioning one's value. Not being seen is painful, but ancient people also had this pain.”

This indicates that loneliness in poetry is not ordinary solitude, but is related to an individual's sense of value, sense of existence, and social position. When readers feel “unseen” during job seeking, career choice, or life lows, poetry provides transhistorical emotional validation. Participants therefore no longer understood their loneliness as an isolated personal problem, but saw that this pain has a more general significance in human experience.

P28 also stated, when discussing Du Fu's Climbing the Heights:

“It did not comfort me directly, but it made me feel that suffering can be acknowledged. After acknowledgment, one can continue.”

This statement shows that the comfort provided by poetry is not always positive encouragement. Some poems are moving precisely because they do not avoid heaviness or rush to dissolve pain, but give pain a space for expression. For readers, the process of being moved by poetry is also a process of acknowledging their own vulnerability and failure.

#### 3.6.3 Subcategory 3: Renewal after hardship and open-minded transcendence

In addition to acknowledging pain, some participants also perceived in poetry a sense of renewal after hardship and an open-minded spirit. This kind of being moved did not come from simple optimism, but from the ability to continue living and to see the world anew after difficult circumstances.

When discussing Liu Yuxi's Replying to Lotte at the First Meeting in Yangzhou, P22 said:

“A damaged stage is not the whole of life. People can keep moving forward with their wounds.”

This quotation indicates that the moving power of “beside the sunken boat, a thousand sails pass; before the diseased tree, ten thousand trees are in spring” lies in the fact that it does not deny injury, but opens a new horizon on the basis of acknowledging injury. What the participant read through the experience of recovering from illness was not empty inspiration, but the possibility of continuing forward with trauma.

P30 also noted Su Shi's Ding Fengbo:

“Open-mindedness is not being naturally optimistic. It is having been beaten by wind and rain, yet still being willing to keep walking.”

This suggests that the open-mindedness readers understood was not ease, absence of pain, or escape from reality, but a spiritual posture formed after weathering storms. The line “neither wind and rain nor clear sky” in Su Shi's lyric moves people precisely because it does not imply that hardship has not been encountered. Rather, after hardship, it regains a distance from which to look at life. Thus, the existential reflection in Tang and Song poetry can provide readers with spiritual settling and give them the strength to continue living amid parting, failure, illness, and impermanence.

### 3.7 Category 6: Barriers and facilitating factors

Tang and Song poetry itself has strong psychological and affective potential, but whether this potential can be felt by readers does not depend entirely on the text itself. It is also influenced by readers‘ life experience, reading context, educational approach, cultural knowledge, and communication environment. Similar life experiences, concrete life situations, oral reading rhythm, and background knowledge can strengthen poetry's moving power. In contrast, exam-oriented reading, labeling, sloganization, distance from allusions, and excessive familiarity may weaken readers' genuine feelings.

#### 3.7.1 Subcategory 1: Facilitating factors

Participants generally believed that poetry's moving power is often strengthened in specific life experiences and concrete life situations. Many lines that were merely recitation material in student years regained emotional force when readers later experienced leaving home, graduation, seeing someone off, heartbreak, illness, failure, or the aging of loved ones.

When discussing Sending Yuan Er on a Mission to Anxi, P01 said:

“Similar experiences strengthen it, such as leaving home, graduating, and seeing someone off at a station.”

When discussing Yulin Ling, P03 stated:

“Real emotional experience, recitation rhythm, and understanding of imagery strengthen it.”

This suggests that the strengthening of poetry's moving power comes not only from personal experience, but also from modes of textual reception. Recitation can strengthen the rhythm and emotional progression of a lyric, while understanding imagery helps readers enter traditional emotional fields such as the long pavilion, willows, and the waning moon. Thus, aesthetic form, cultural knowledge, and personal experience work together to make readers more likely to be moved by poetry.

In addition, authorial background and historical situation can also strengthen the experience of being moved. When discussing Liu Yuxi's Replying to Lotte at the First Meeting in Yangzhou, P22 said:

“Knowing that Liu Yuxi was banished for many years strengthens the feeling. It is not pretty words spoken from favorable circumstances, but something said after suffering.”

This quotation indicates that background knowledge can enhance the emotional credibility of poetry. When readers know that a line emerged from long-term banishment, recovery from illness, or life frustration, the open-mindedness in the poem is no longer empty encouragement, but a genuine expression after hardship.

#### 3.7.2 Subcategory 2: Weakening factors

In contrast to facilitating factors, some participants also noted that certain modes of reception can weaken the moving power of Tang and Song poetry. The most prominent among these were exam-oriented and standard-answer approaches. In school education, poetry is often transformed into fixed knowledge points, causing readers to remember only “what thought and feeling it expresses” while neglecting their own genuine feelings.

P01 said:

“What weakens it is exam-oriented reading. If one only memorizes that it ‘expresses reluctant farewell,' it is very hard to be truly moved.”

This suggests that standardized interpretation may conceptualize and formulaize poetic emotion. Readers may be able to answer the theme of a poem, but may not truly enter the scene and emotion within it.

P31 offered a similar view:

“If only standard answers are taught, students cannot feel the bodily pain in the poem.”

This statement further indicates that poetry education weakens poetic emotional intensity when it overemphasizes conclusions while neglecting concrete situations, bodily feelings, and life experience. This is especially true for poems about family-country concern, life frustration, parting, and longing. If they are summarized only as “concern for the country and the people,” “reluctant farewell,” or “homesickness,” their complex and genuine pain is easily obscured.

In addition, distance from allusions and excessive familiarity may also weaken poetry's moving power. When discussing Wuyi Lane, P20 stated:

“What weakens it is distance from allusions, not knowing what rise and fall it is writing about.”

This shows that for works that rely heavily on historical allusions, authorial experience, and cultural background, readers often find it difficult to enter their deeper emotional structure without necessary knowledge support. At the same time, some widely circulated famous poems and lines may also undergo aesthetic dulling because of repeated recitation, frequent quotation, and formulaic interpretation, weakening what was originally a fresh emotional experience.

## 4 Discussion

From the perspective of the aesthetics of reception, this study used semi-structured interviews and inductive content analysis to examine the experiential structure and interpretive psychological processes through which Tang and Song poetry moves contemporary readers. Six major categories and 20 subcategories were extracted from the interview data: sensory aesthetics, individual experience, relational emotions, cultural memory, existential reflection, and barriers and facilitating factors. The results show that Tang and Song poetry continues to move contemporary readers not merely because of beautiful language or elevated artistic conception, but because of complex interactions among textual form, reader experience, relational memory, cultural accumulation, and life understanding. In other words, being moved is not an effect unilaterally imposed by the text on the reader, but a composite subjective experiential process formed through readers' acts of reading, remembering, associating, and self-understanding.

Across the six categories, being moved can be understood as an open interpretive process rather than a fixed sequence. Language, rhythm, action, and imagery provide aesthetic triggers. Self-reference and autobiographical memory connect the text with individual experience. Family affection, friendship, love, and family-country relations give emotions a relational direction. Educational experience, traditional imagery, and historical knowledge provide a cultural framework for understanding. Readers then interpret the life meanings of parting, impermanence, failure, and renewal. Readers may enter at any point and move back and forth among these components, so the process is not a linear causal chain. Theoretically, textual openness corresponds to Iser's structure of appeal, cultural expectations to Jauss's horizon of expectations, and the co-construction of text with personal and relational experience to Rosenblatt's transactional reading. Aesthetic psychology links perception, emotion, memory, and meaning construction.

This study found that sensory aesthetics is the basic entry point through which Tang and Song poetry moves readers. Participants were often first attracted by condensed and precise language, action details, imagery, visual scenes, and multisensory atmosphere, and only then entered personal experience and reflection on life. Existing research in the psychology of poetry shows that formal features such as meter and rhyme can influence readers' aesthetic evaluation and emotional involvement (Obermeier et al., 2013), and that aesthetic experience is closely related to processing fluency and readers' emotional responses (Reber et al., 2004). This finding is also consistent with the aesthetics of reception and reader-response theory: literary meaning does not exist as a fixed entity within the text, but is realized in the interaction between textual structure and reader experience. The text guides readers‘ participation in meaning generation through blanks, expectations, and structures of appeal (Iser, 1978; Jauss, 1982). Importantly, textual features do not produce effects independently. The same imagery, rhythm, or linguistic structure is more likely to evoke deep resonance when it matches readers' prior experience, relational memory, and cultural knowledge. Conversely, unfamiliar allusions, standardized interpretation, or excessive familiarity may weaken this connection.

Second, individual experience is a key condition for activating poetry's moving power. Many participants mentioned that they mechanically recited poems during their student years, and that genuine being moved often occurred only after leaving home, living elsewhere, heartbreak, failure, illness, or aging. This indicates that the meaning of Tang and Song poetry has a delayed mode of emergence. According to the aesthetics of reception and transactional reading theory, literary meaning is not transmitted one-way by the text, but is generated in the interaction between textual structure and reader experience (Rosenblatt, 1978). Research on autobiographical memory and self-referential processing also shows that individuals are more likely to engage in deep processing and emotional involvement with information related to their own experiences, identity, and life stage (Conway and Pleydell-Pearce, 2000; Symons and Johnson, 1997). Thus, readers do not necessarily obtain a deep experience at first contact with a text. Rather, they re-understand poetry in later life situations. Themes such as homesickness, drifting, frustration, and aging often require corresponding life experience before they can be truly perceived. The moving power of poetry is therefore not fixed, but is continually reactivated as readers' life stages, emotional states, and self-understanding change.

Third, relational emotions are an important mediator of how Tang and Song poetry moves readers. The study found that participants rarely understood emotion in poetry as isolated individual feeling. Instead, they often placed it within relationships of family affection, friendship, love, marriage, and family-country belonging. Social psychology has shown that establishing and maintaining social connections is a fundamental human psychological need, and that individuals' emotional experiences are often embedded in concrete interpersonal relationships and social relational structures (Baumeister and Leary, 1995). Literary reading is also thought to enhance readers' social-emotional experience by simulating social experience, activating relational imagination, and promoting understanding of others' situations (Mar and Oatley, 2008). For example, homesickness ultimately points to parents and family; farewell ultimately points to a friend's departure; longing ultimately points to attachment in intimate relationships; and family-country concern ultimately points to the vulnerability of ordinary families during historical upheaval. This indicates that the emotions in Tang and Song poetry are distinctly relational. They do not merely express the emotion of “I,” but move readers through relational structures of “I and others,” “I and hometown,” and “I and the times.”

In addition, cultural memory played a dual role in poetic reception. On the one hand, textbook education, traditional imagery, and historical allusions provide readers with a basis for understanding poetry, enabling poems to be preserved in collective memory over the long term and reawakened in appropriate life situations. Cultural memory theory holds that classical texts, ritualized transmission, and educational systems jointly maintain a group's shared memory and cultural identity (Assmann, 2011; Erll, 2011). On the other hand, exam-oriented, standard-answer, and label-based modes of reception may weaken poetry's genuine moving power. When poems are reduced to fixed answers such as “expressing homesickness” or “embodying concern for the country and the people,” readers can easily lose sensitivity to concrete situations, bodily feelings, and emotional details. Transactional reading theory emphasizes that literary teaching should not focus only on extracting fixed meanings, but should also value the active interaction between readers' personal experience and the text (Rosenblatt, 1978). Therefore, cultural education does not necessarily weaken poetic experience. The key is whether education can help readers connect textual knowledge with genuine experience.

The study also found that Tang and Song poetry can provide readers with existential reflection and spiritual settling. Some participants stated that poetry does not necessarily solve real problems directly, but can help them acknowledge parting, loneliness, failure, impermanence, and suffering. Meaning-making theory suggests that when individuals face stress, loss, and life adversity, they often need to re-understand their situation through narratives, symbols, and interpretive frameworks (Park, 2010). Research on aesthetic emotions also shows that sadness, being moved, sublimity, and complex emotions in literature and art do not necessarily produce negative experience. Instead, they may help individuals achieve emotional integration and a sense of meaning (Menninghaus et al., 2019). Especially when discussing works such as Climbing the Heights, Song on Climbing Youzhou Terrace, Replying to Lotte at the First Meeting in Yangzhou, and Ding Fengbo, participants did not feel only sadness or open-mindedness, but obtained from them an understanding of life's situations. The comfort provided by poetry is not always positive encouragement. Sometimes it simply allows pain to be spoken, seen, and acknowledged. This feeling of being understood constitutes an important experiential process through which Tang and Song poetry moves contemporary readers.

It is worth noting that the category of barriers and facilitating factors in this study suggests that the moving power of Tang and Song poetry does not occur automatically. Similar life experiences, concrete reading situations, recitation rhythm, knowledge of traditional imagery, and understanding of authorial background may all strengthen readers‘ experience of being moved. Conversely, excessive familiarity, distance from allusions, exam-oriented interpretation, sloganized explanation, and inspirationalized dissemination may weaken poetry's affective power. This finding has implications for the contemporary reception and communication of classical poetry. If Tang and Song poetry is to truly move contemporary readers, teaching and dissemination should not emphasize only recitation, thematic summary, and standard interpretation. They should also guide readers to connect poetry with their own experiences and to enter the concrete situations and emotional structures within the poems. In process terms, similar life experiences increase experiential matching; recitation rhythm and concrete settings strengthen attentional engagement; and knowledge of imagery and allusions improves textual accessibility. Conversely, unfamiliar allusions reduce comprehensibility, exam-oriented or standardized interpretation constrains readers' active meaning generation, and excessive familiarity diminishes attention and novelty. Facilitating factors and barriers therefore condition the other categories rather than form an independent outcome pathway. Theoretically, this study specifies the text-reader interaction proposed by the aesthetics of reception as an interpretive structure comprising aesthetic triggering, experiential connection, relational and cultural mediation, meaning construction, and conditional modulation. It thereby adds reader-response evidence from the context of classical Chinese poetry. In practice, poetry education and public cultural communication may use contextualized recitation, links to personal experience, scaffolding for imagery and allusions, and open-ended discussion. These approaches are intended to improve textual accessibility and reader engagement and should not be interpreted as clinical interventions.

This study has several limitations. First, it included both Tang poetry and Song ci. Although this helped reveal shared experiential processes through which classical Chinese poetry moves contemporary readers, it may also have weakened differences among genres, themes, and historical contexts. Recruitment through universities, communities, online platforms, and acquaintance referrals may also have introduced self-selection bias by favoring participants who were willing to articulate their experiences or already familiar with poetry. Future research could focus further on specific text types, such as Song ci, farewell poems, homesickness poems, or mourning lyrics, to compare differences in aesthetic arousal, emotional resonance, and meaning construction across poetic forms. Second, this study used qualitative interviews and relied mainly on participants‘ retrospective narratives. The results may therefore be influenced by personal memory, expressive ability, and current emotional state, and cannot directly support causal inferences between poetry reading and psychological responses. Third, participants' age, educational background, familiarity with classical literature, and prior reading experience may all influence their reception experience. Future studies could use stricter stratified sampling and combine quantitative surveys or experimental designs for further testing. Moreover, because participants selected their own works and did not receive a standardized poetic stimulus, the study cannot compare the relative effects of Tang poetry and Song ci. Here, a ‘shared process' refers only to experiential structures that recurred across narratives about different texts. It does not imply that Tang poetry and Song ci operate in the same manner or with the same strength. Genre differences should be tested in future studies using paired texts or stratified designs.

## 5 Conclusion

Based on the aesthetics of reception, this study aimed to examine the experiential structure and interpretive psychological processes through which Tang and Song poetry moves contemporary readers. The findings provide a new perspective for understanding the aesthetic reception, emotional resonance, and cultural meaning of classical poetry in contemporary contexts. The results show that the continuing power of Tang and Song poetry to move readers does not arise only from the linguistic beauty or elevated artistic conception of the text itself, but is generated in the encounter between textual form and reader experience.

The study found that Tang and Song poetry moves readers mainly in six ways: (i) at the level of sensory aesthetics, condensed and precise language, concrete action details, imagery and visual scenes, and multisensory experience provide readers with an aesthetic entry point into poetry; (ii) at the level of individual experience, experiences such as leaving home and drifting, waiting in love, life frustration, aging, and illness reactivate poems that were once familiar; (iii) at the level of relational emotions, family affection, friendship, love, marriage, and family-country relations give the emotions in poetry concrete interpersonal direction; (iv) at the level of cultural memory, textbook education, traditional imagery, authorial background, and historical allusions provide readers with a shared cultural framework for understanding poetry; (v) at the level of existential reflection, poetry helps readers accept parting, impermanence, loneliness, failure, and suffering, and to some extent gain spiritual settling; and (vi) at the level of influencing factors, similar experiences, concrete situations, recitation rhythm, and background knowledge can strengthen poetry's moving power, whereas exam-oriented reading, labeling, sloganization, and distance from allusions may weaken it. Textual aesthetics provides an entry point; individual experience activates meaning; relational emotions deepen resonance; cultural memory provides an interpretive framework; existential reflection enables spiritual settling; and concrete conditions of reception influence whether this moving power can fully occur.

Tang and Song poetry still has important aesthetic, emotional, psychological, and cultural value for contemporary readers. These poems do not exist only as static literary classics. Rather, during reading, they continuously participate in individuals‘ emotional arousal, activation of self-memory, self-understanding, relational recollection, and construction of cultural identity. From this perspective, studying why Tang and Song poetry moves people not only clarifies the interpretive psychological processes involved in the contemporary reception of classical Chinese poetry, but also provides empirical insight into how classical cultural texts participate in emotional expression, meaning construction, relational understanding, and cultural belonging. This study contributes to research on aesthetic emotions by extending being moved from general art reception to the context of classical Chinese poetry, showing that it is not a single form of aesthetic pleasure, but a composite psychological experience jointly generated by textual form, self-memory, relational emotions, cultural schemas, and existential meaning. These findings reflect participants' reported subjective experiences and do not constitute evidence that poetry reading produces health or clinical effects.

## Data Availability

The raw data supporting the conclusions of this article will be made available by the authors, without undue reservation.
